# Supplementary material for: Chiral Amphiphilic Secondary Amine-Porphyrin Hybrids for Aqueous Organocatalysis
Source: Molecules. 2020 Jul 28;25(15):3420. doi: 10.3390/molecules25153420 (PMC7435841; doi:10.3390/molecules25153420)
Supplement: Supplementary file 1 [file molecules-25-03420-s001.pdf]

Molecules
Supplementary Material:

Electronic Supporting Information for

Chiral Amphiphilic Secondary Amine-Porphyrin Hybrids for Aqueous Organocatalysis

Aitor Arlegui, Pol Torres, Victor Cuesta, Joaquim Crusats*, and Albert Moyano*

Departament de Química Inorgànica i Orgànica, Secció de Química Orgànica; Institut de Ciències del Cosmos (ICC), Universitat de Barcelona, Martí Franquès 1, 08028-Barcelona, Catalonia, Spain

SUMMARY

- Chemical structures of the sulfonated porphyrins. (p. 2)
- HPLC chromatograms of the water-soluble sulfonated porphyrins. (p. 3)
- Spectrophotometric determination of the pK_a values of compounds **8** and **13**. (p. 4-5)
- Effect of the porphyrin concentration on their aggregation behaviour. (p. 6)
- ^{1}H and ^{13}C NMR spectra of new compounds. (p. 7-12)
- HPLC traces of Michael and aldol adducts. (p. 13-18)

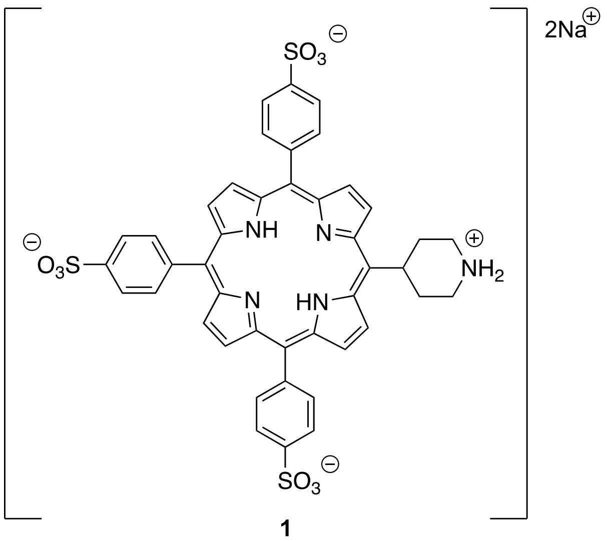

1

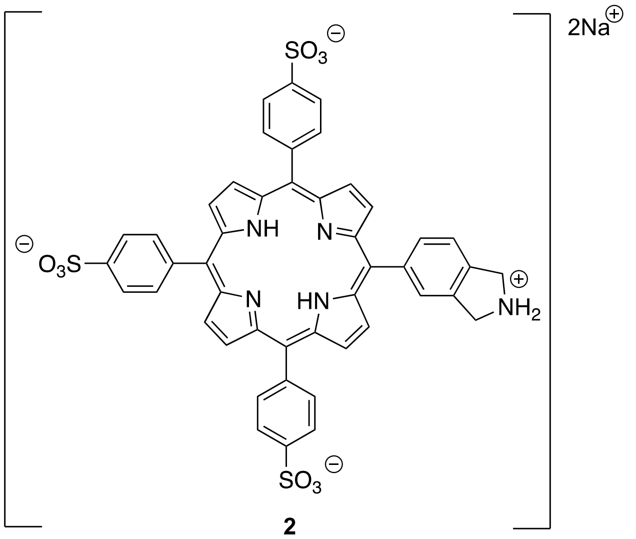

2

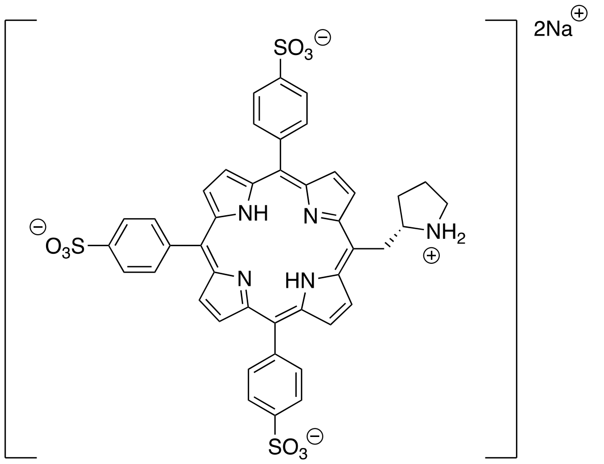

8

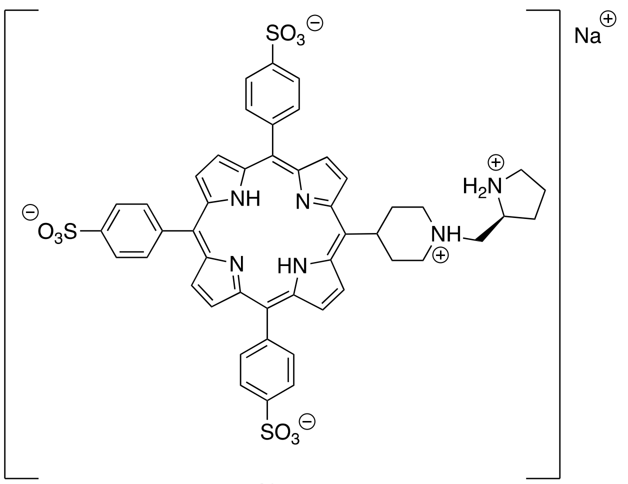

13

Chart ESI-1. Chemical structures of the sulfonated porphyrins **1**, **2**, **8**, and **13** (neutral pH)

HPLC analysis of the sulfonated porphyrins

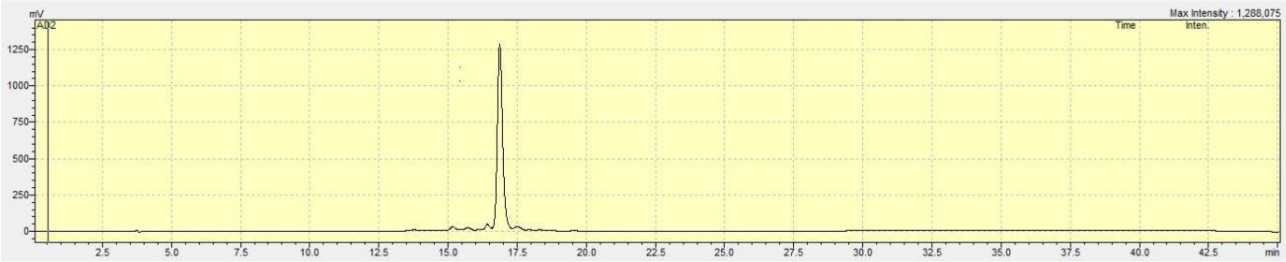

Porphyrin **8**

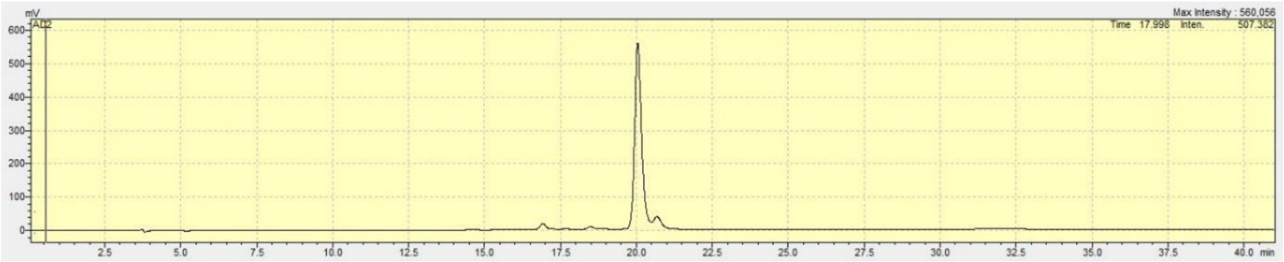

Porphyrin **13**

Figure ESI-1. Reverse-phase analytical HPLC chromatograms for the water-soluble sulfonated porphyrins **8** and **13**. The chromatographic conditions are described in the Experimental section of the main text. The porphyrins had been previously desalted and enriched by medium pressure column chomatography using MCI GEL® CHP20P 75-150 μm. The small bands in front of the main one correspond to small amounts of the products in which sulfonation has taken place on the *meta*-phenylic position of the substituent. Under the experimental conditions of sulfonation reported in the main text, partially sulfonated porphyrins bearing only two sulfonato groups were not detected in any case.

Spectrophotometric determination of the pK_a values of the sulfonated porphyrins 8 and 13

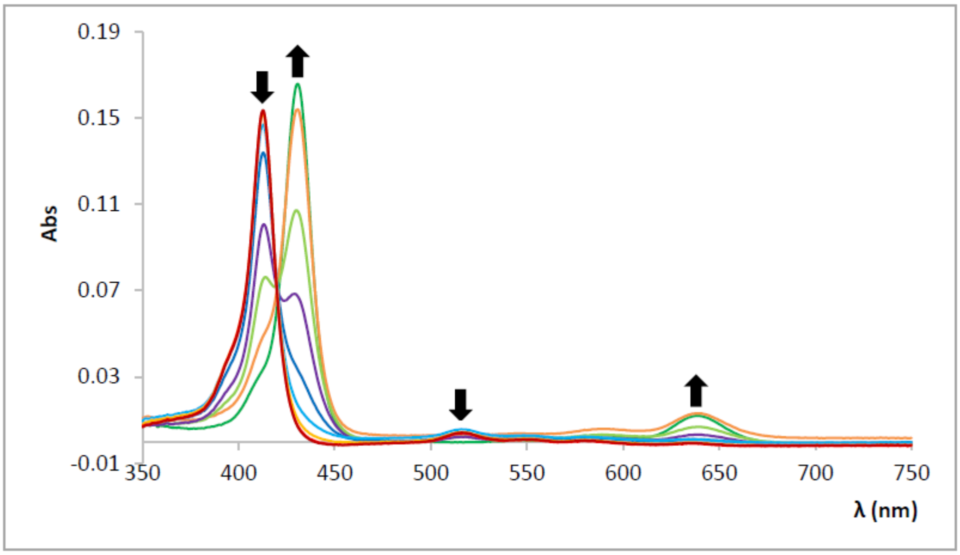

Figure ESI-2. Spectrophotometric titration (cell path 1 cm) of a 3.67 $\cdot 10^{-7}$ M aqueous solution of porphyrin **8** (see the quantitative details on the next pages). The arrows show the spectral changes recorded at different pH values in 0.1 M AcOH/AcONa buffers from solutions in which the porphyrins are 100% in their free-base form to those in which they are 100% in their diacidic form (both inner core pyrroleninic protons being protonated). The pH values of the solutions are: 7.89, 6.14, 5.48, 4.99, 4.46, 4.01, 3.66, 3.34, and 2.78.

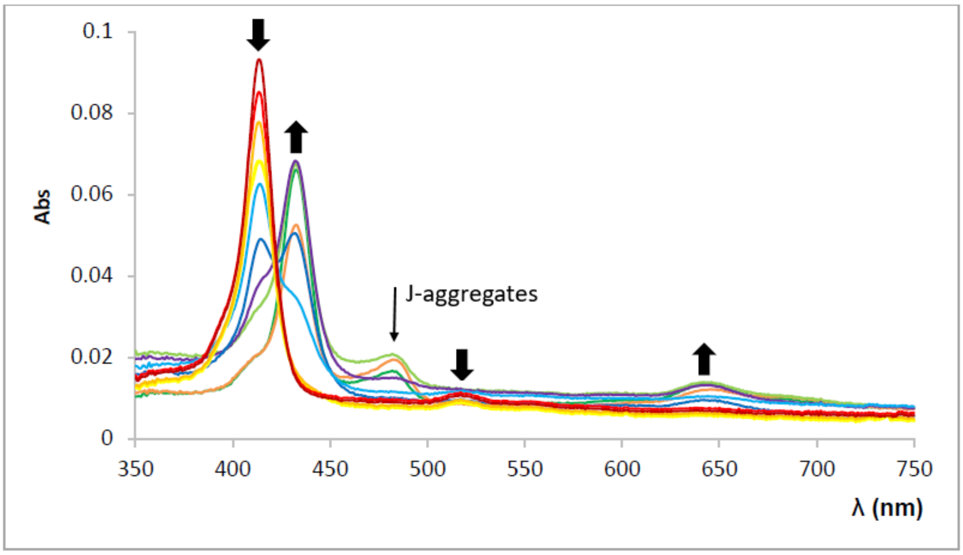

Figure ESI-3. Spectrophotometric titration (cell path 1 cm) of a 1.51 $\cdot 10^{-7}$ M aqueous solution of porphyrin **13** (the formation of J-aggregates, even in these highly diluted conditions, when the deprotonated species predominates preclude the exact determination of the pK_a value of this porphyrin). The arrows show the spectral changes recorded at different pH values in 0.1 M AcOH/AcONa buffers from solutions in which the porphyrins are 100% in their free-base form to those in which they are 100% in their diacidic form (both inner core pyrroleninic protons being protonated). The pH values of the solutions are: 7.91, 6.00, 5.60, 5.20, 4.80, 4.39, 3.99, 3.60, 3.19, and 2.60.

Table ESI-1. Numerical values obtained from the spectra in Figure ESI-2 (porphyrin **8**).

pH	Abs (413 nm)	$\log_{10}\frac{A - A_{base}}{A_{acid} - A}$	Abs (431 nm)	$\log_{10}\frac{A - A_{base}}{A_{acid} - A}$
2.78	0.033994	100% diacidic form	0.165769	100% diacidic form
3.34	0.049242	0.83520873	0.153895	1.09360003
3.66	0.075521	0.27409367	0.106573	0.22758073
4.01	0.100709	-0.10100422	0.067138	-0.21200702
4.46	0.134000	-0.70820139	0.032972	-0.70209211
4.99	0.146976	-1.23315486	0.016623	-1.17270022
5.48	0.151677	-1.79113613	0.010077	-1.65145539
6.14	0.152969	-2.28913476	0.007101	-2.50335173
7.89	0.153580	100% free base	0.006603	100% free base

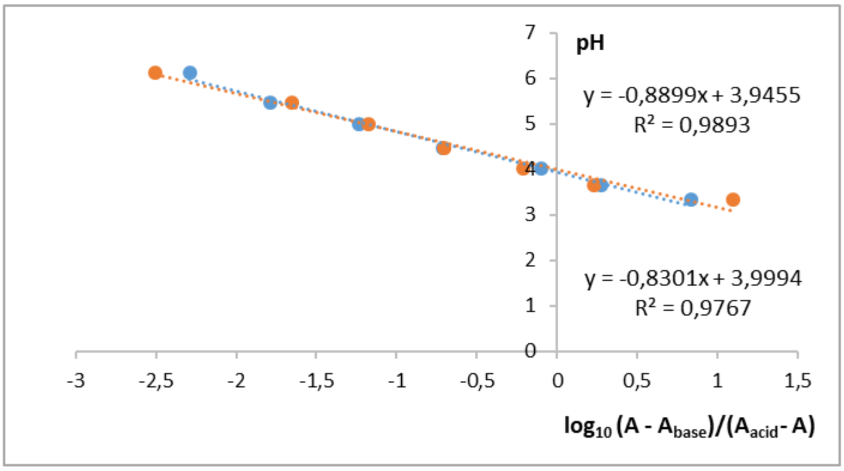

The reported pK_a values correspond to the average of those determined at the maximum absorption wavelengths of the free-base species and of the diacidic form, and could be consistently reproduced within the experimental error when the titrations were perfomed with new freshly prepared solutions.

pK_a of porphyrin **8**: 3,97 ±0.02

Owing to the presence of J-aggregates even in highly diluted solutions of porphyrin **13** (see Figure ESI-3) only an estimated pK_a value of 4.39 could be obtained for this compound.

Effect of the porphyrin concentration on their aggregation behaviour

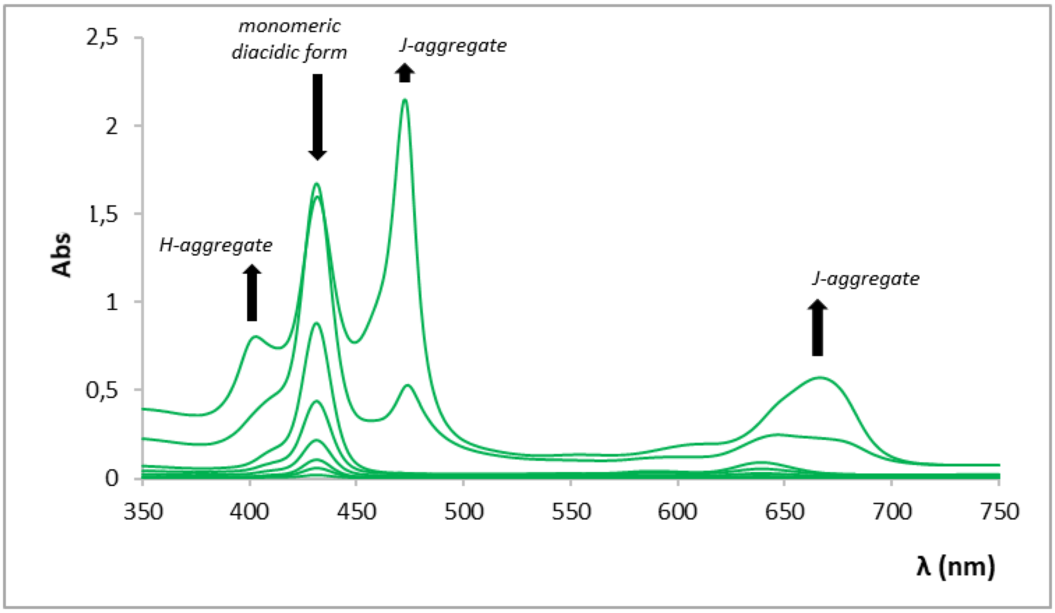

Figure ESI-4. UV-vis spectra (cell path 1 mm) of porphyrin **8** at increasing concentrations of freshly prepared solutions in HCl 0.1 M: $4.80 \cdot 10^{-7}$ M, $2.36 \cdot 10^{-6}$ M, $4.72 \cdot 10^{-6}$ M, $9.44 \cdot 10^{-6}$ M, $1.89 \cdot 10^{-5}$ M, $3.78 \cdot 10^{-5}$ M, $7.56 \cdot 10^{-5}$ M, and $1.11 \cdot 10^{-4}$ M.

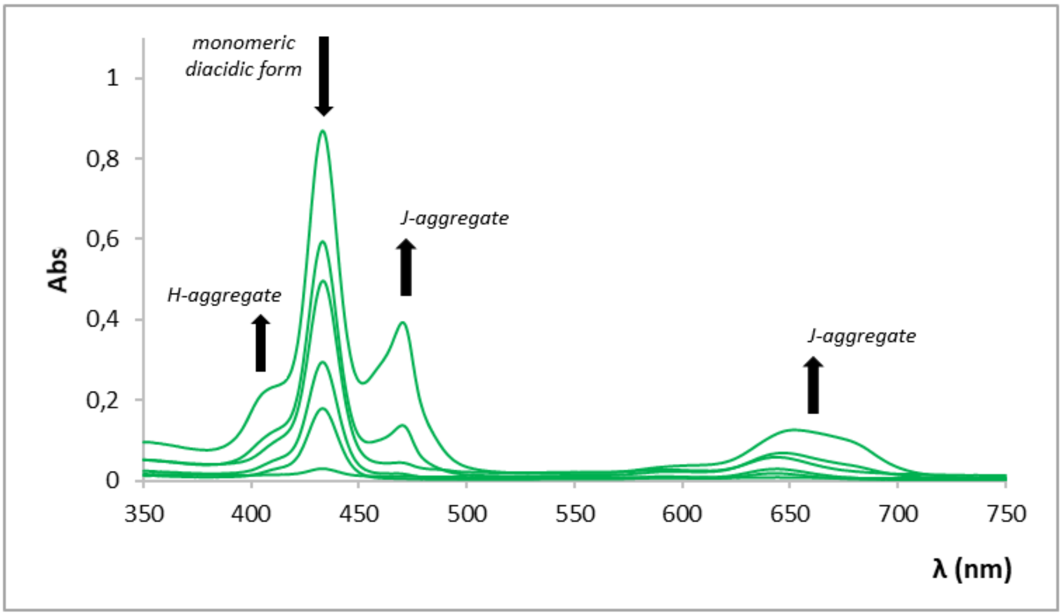

Figure ESI-5. UV-vis spectra (cell path 1 mm) of porphyrin **13** at increasing concentrations of freshly prepared solutions in HCl 0.1 M: $1.00 \cdot 10^{-7}$ M, $1.00 \cdot 10^{-6}$ M, $1.75 \cdot 10^{-6}$ M, $2.50 \cdot 10^{-6}$ M, $5.00 \cdot 10^{-6}$ M, and $1.00 \cdot 10^{-5}$ M.

(*S*)-5-[(*N*-Boc-pyrrolidin-2-yl)methyl]-10,15,20-triphenylporphyrin (7)

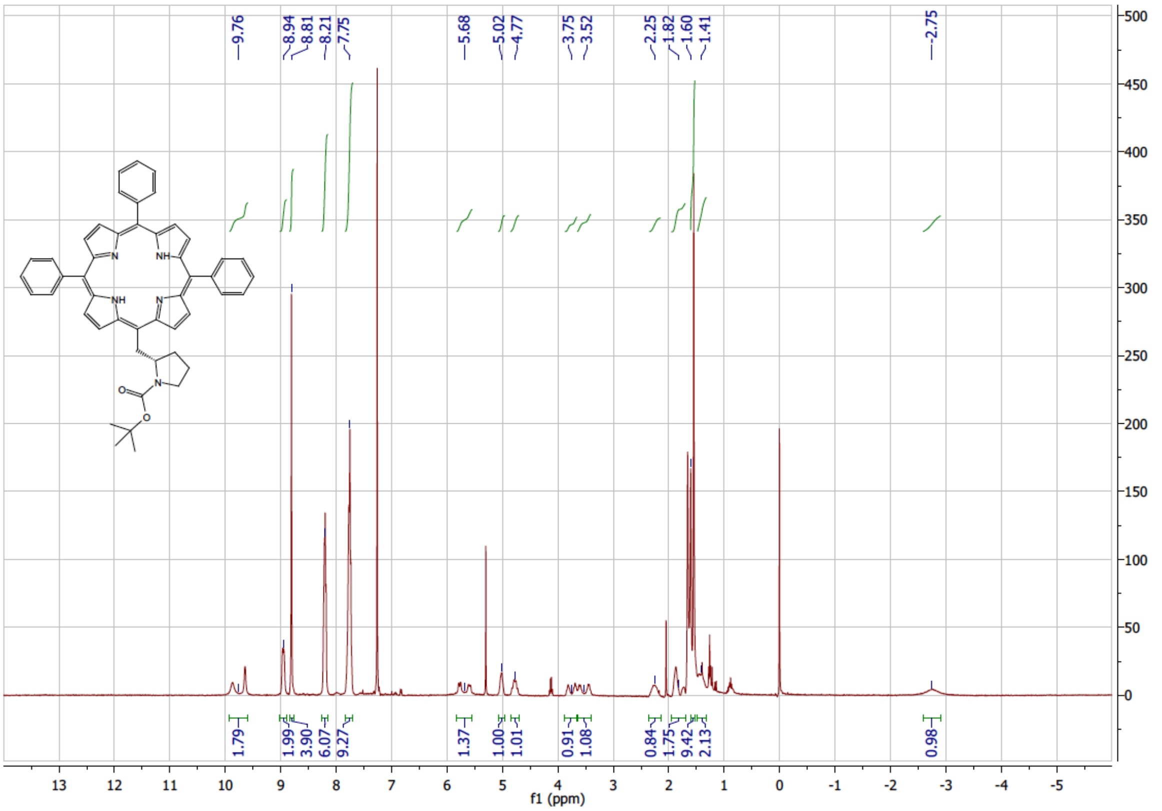

^{1}H NMR (400 MHz, $CDCl_3$, TMS_{int})

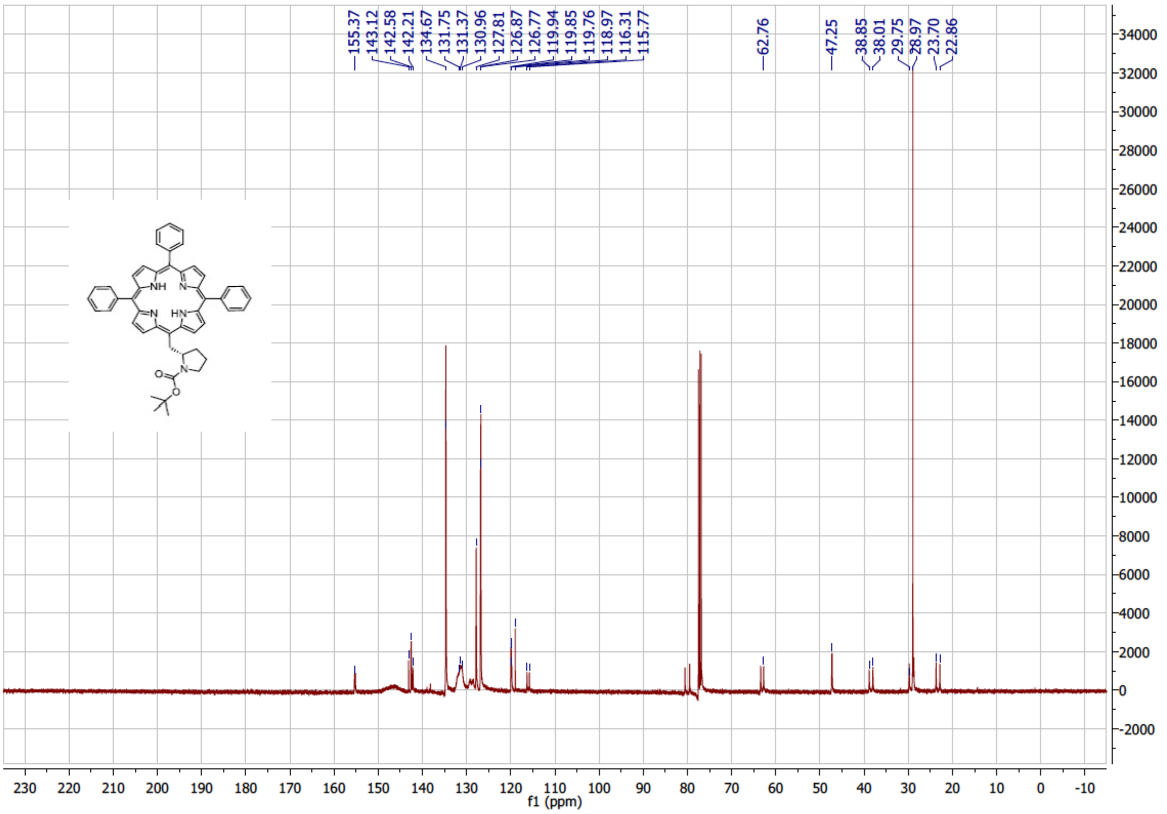

^{13}C NMR (100 MHz, $CDCl_3$)

N-Boc-(*S*)-2-[(4-(hydroxymethyl)piperidin-1-yl)methyl]pyrrolidine (10)

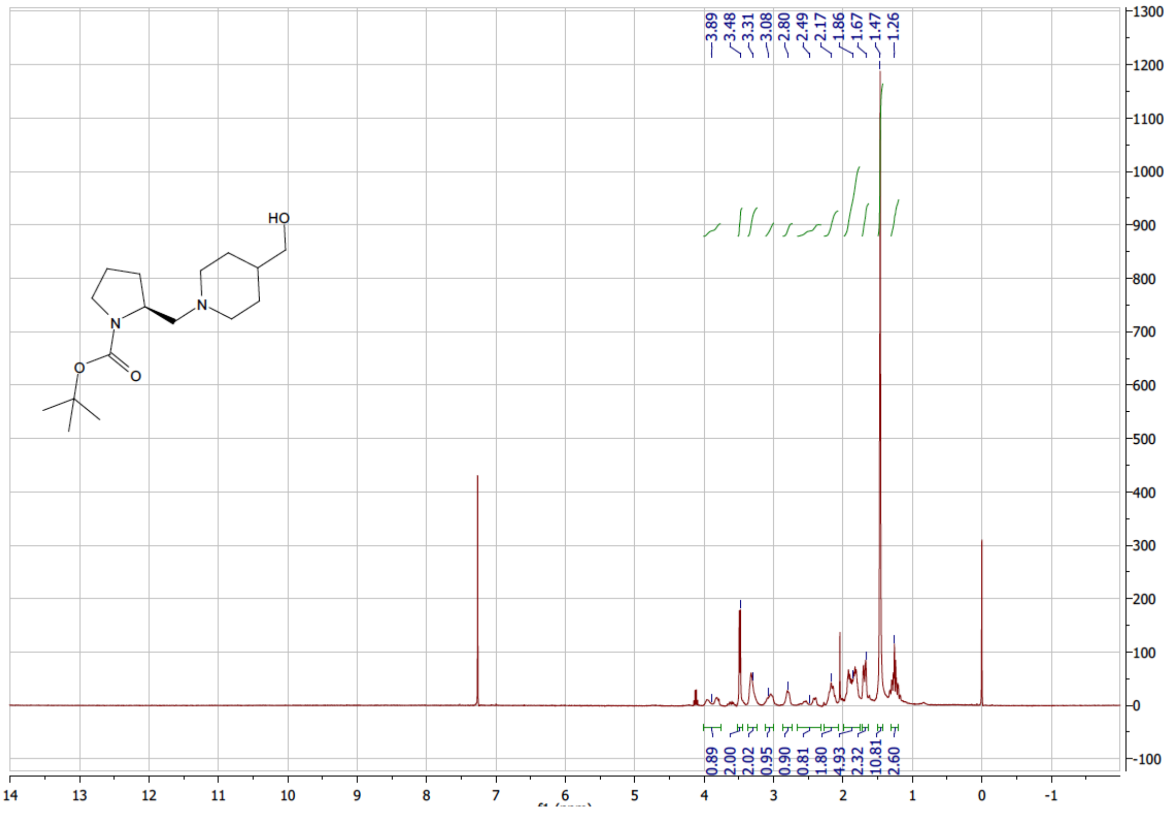

^{1}H NMR (400 MHz, $CDCl_3$, TMS_{int})

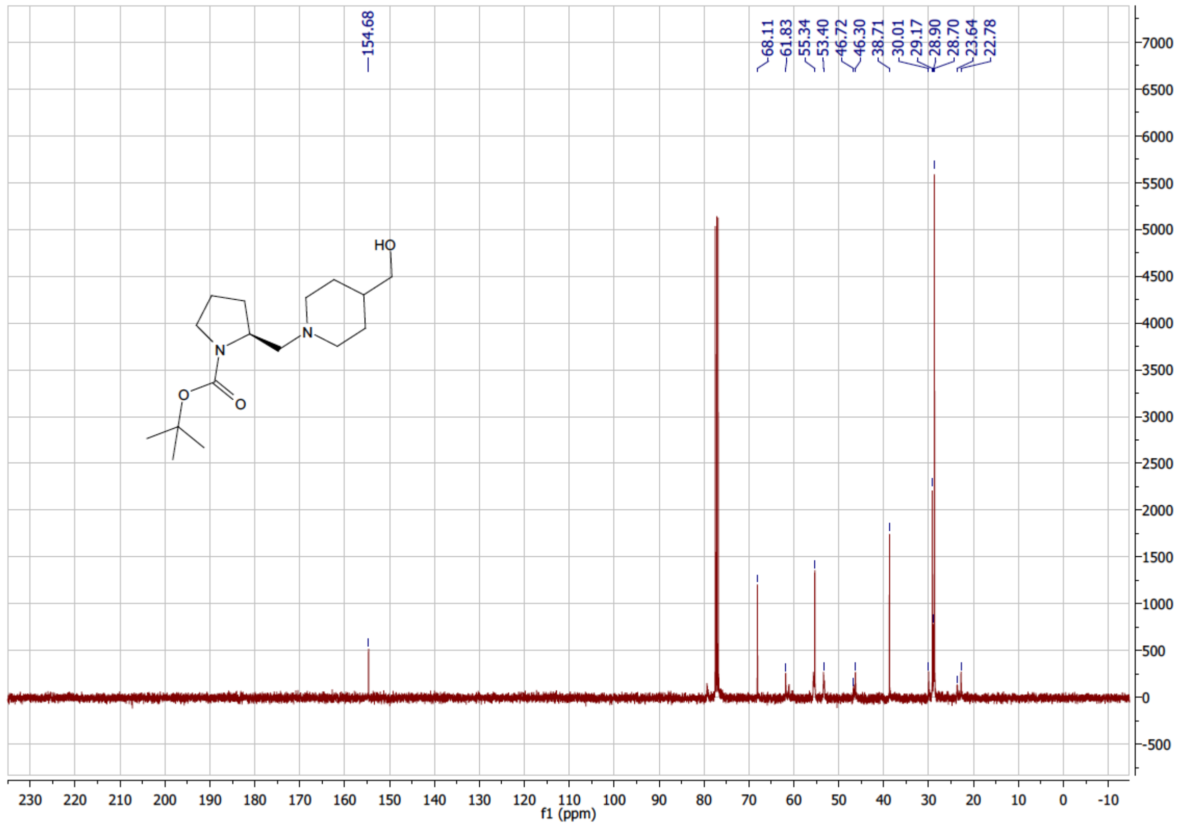

^{13}C NMR (100 MHz, $CDCl_3$)

_N_-Boc-(_S_)-2-[(4-formyl-piperidin-1-yl)methyl]pyrrolidine (11)

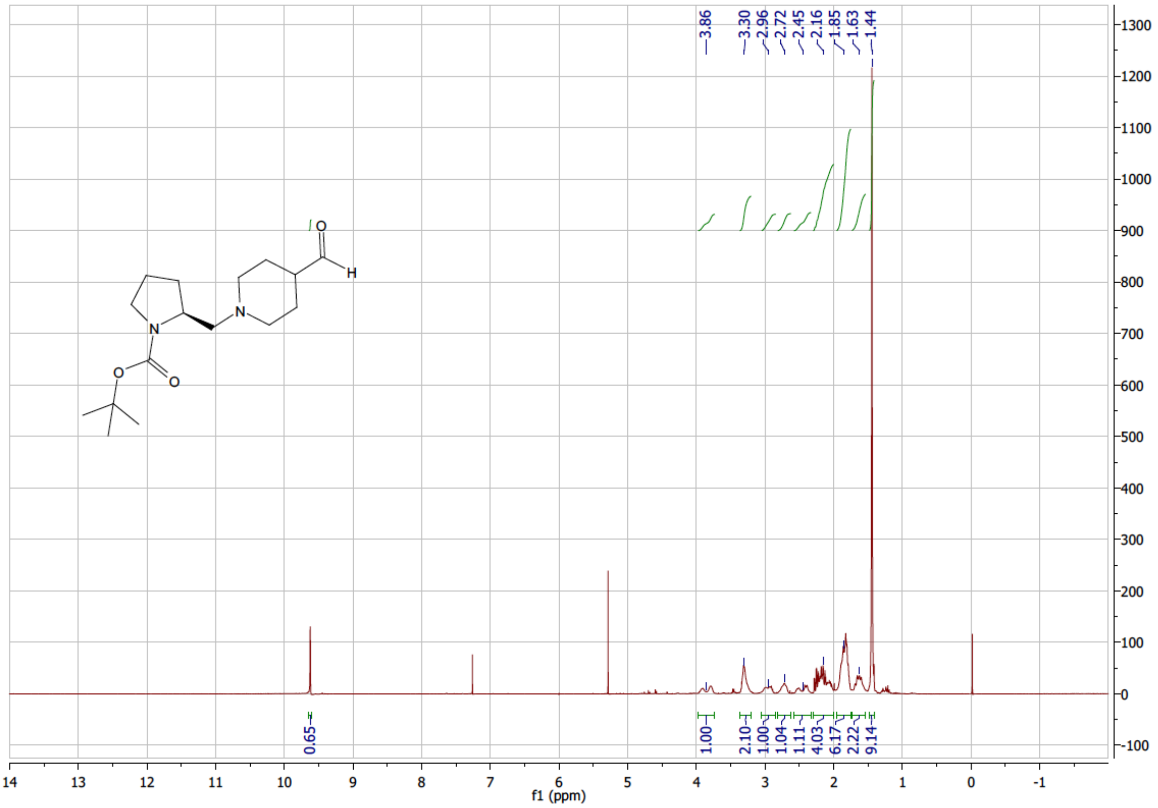

^{1}H NMR (400 MHz, $CDCl_3$, TMS_{int})

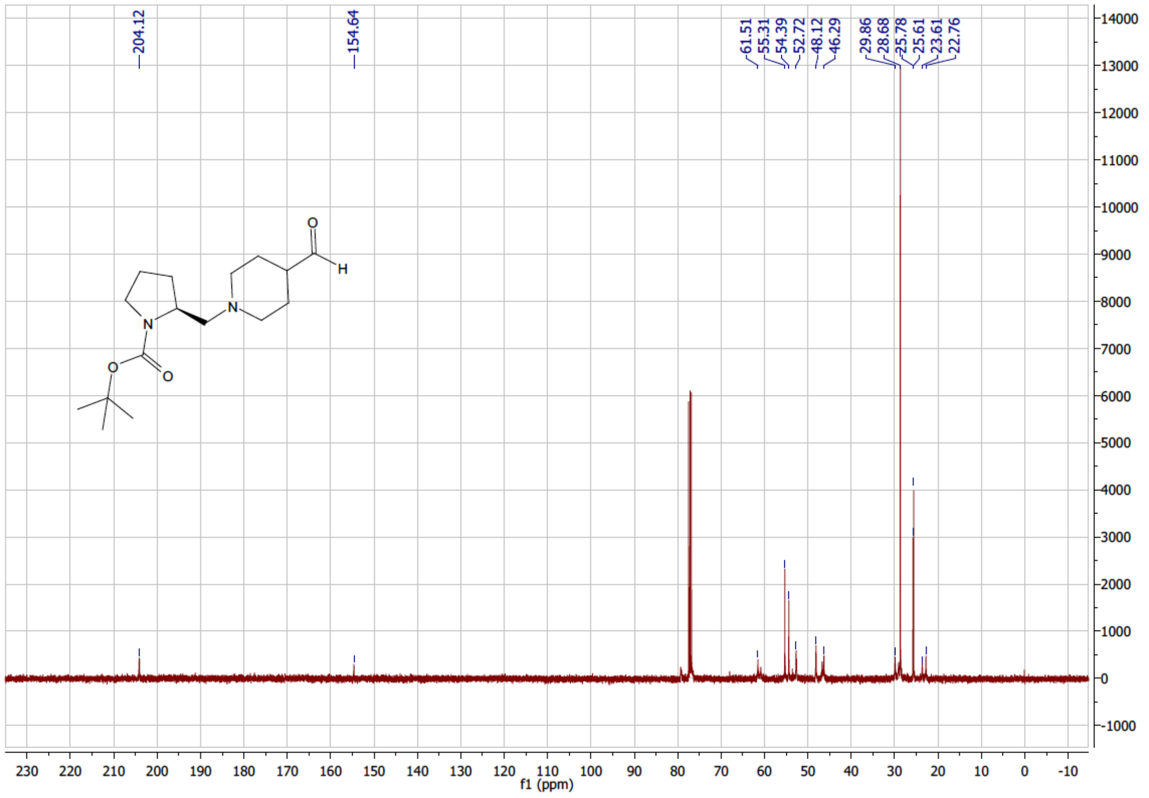

^{13}C NMR (100 MHz, $CDCl_3$)

(*S*)-5-[1-*N*-Boc-2-(methylpyrrolidinyl)piperidin-4-yl]-10,15,20-triphenylporphyrin (14)

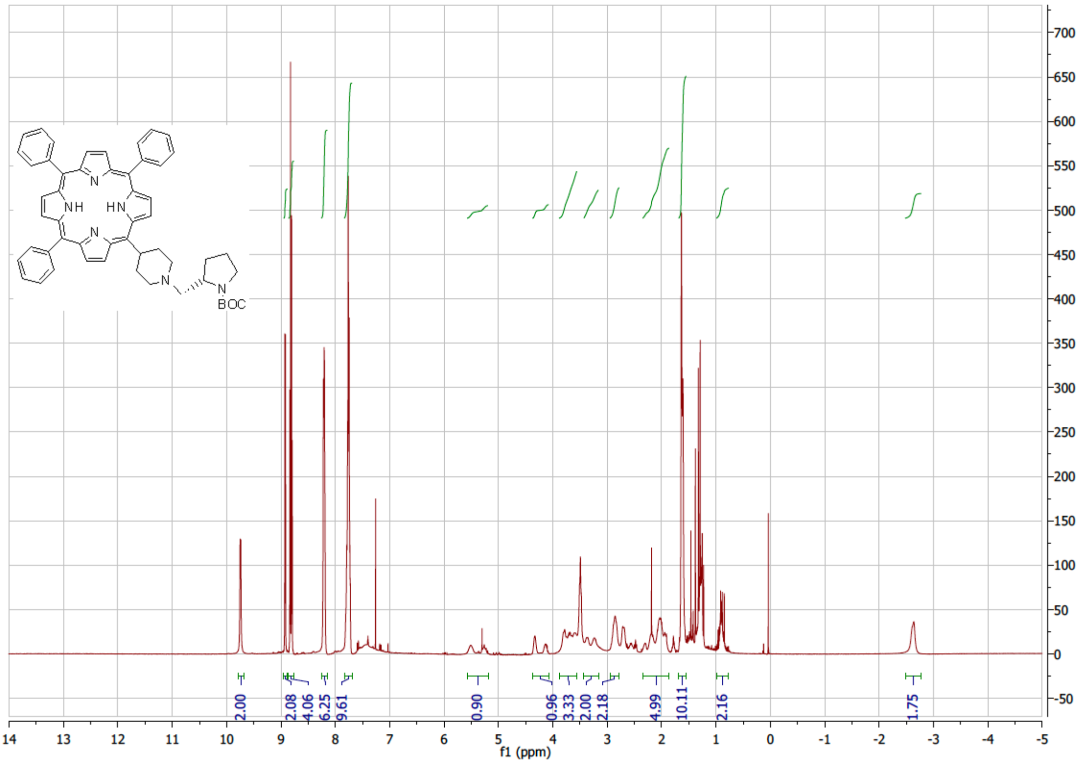

^{1}H NMR (400 MHz, $CDCl_3$, TMS_{int})

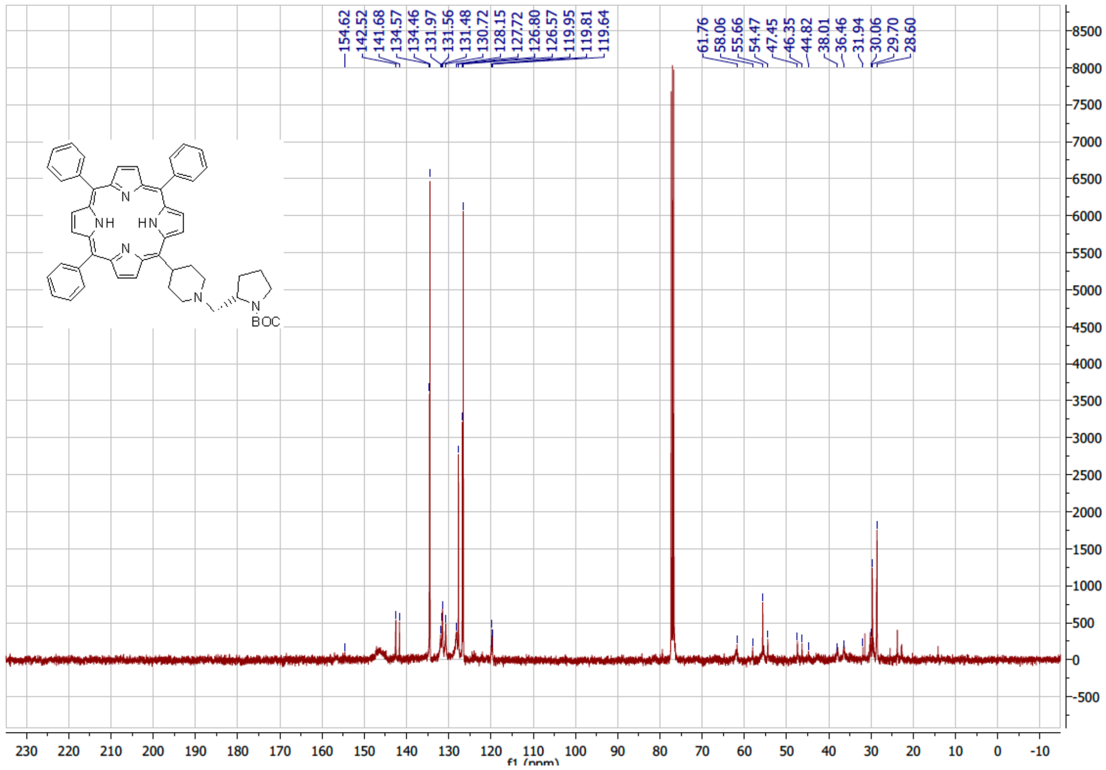

^{13}C NMR (100 MHz, $CDCl_3$)

(*S*)-5-[(Pyrrolidin-2-yl)methyl]-10,15,20-tris(4-sulfonatophenyl)porphyrin trisodium salt (8)

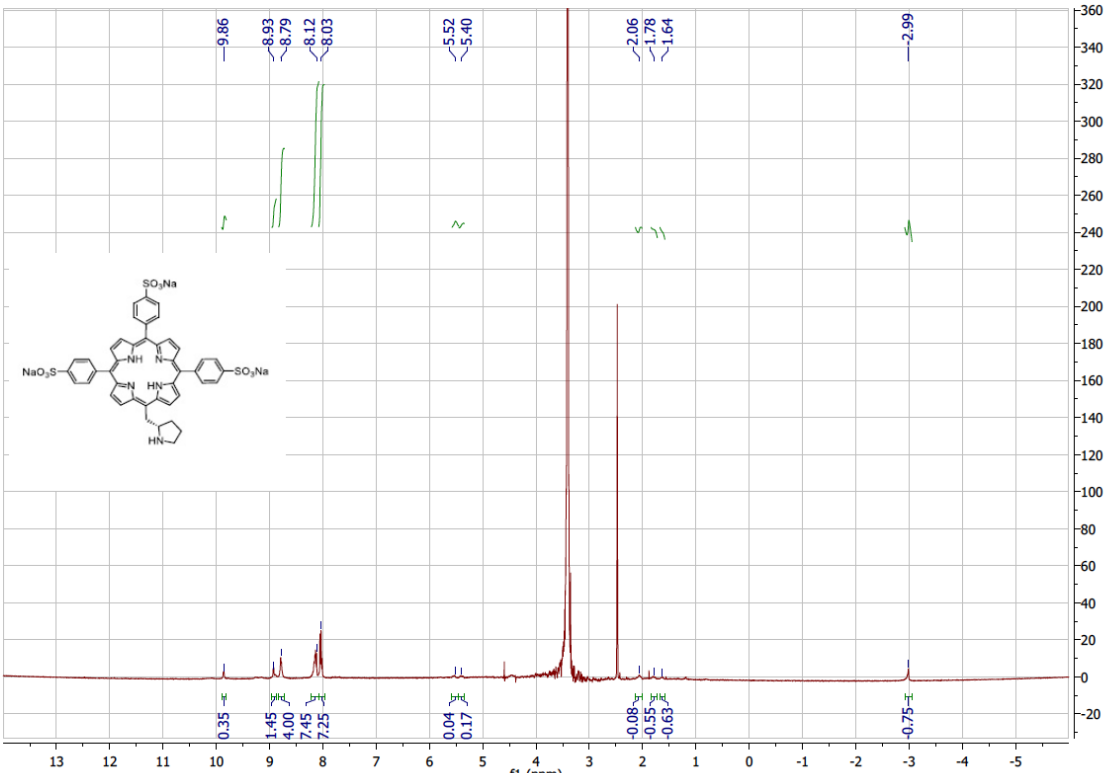

^{1}H NMR (400 MHz, DMSO-d_6, TMS$_{int}$)

(*S*)-(1-(Pyrrolidin-2-ylmethyl)piperidin-4-yl)methanol (17)

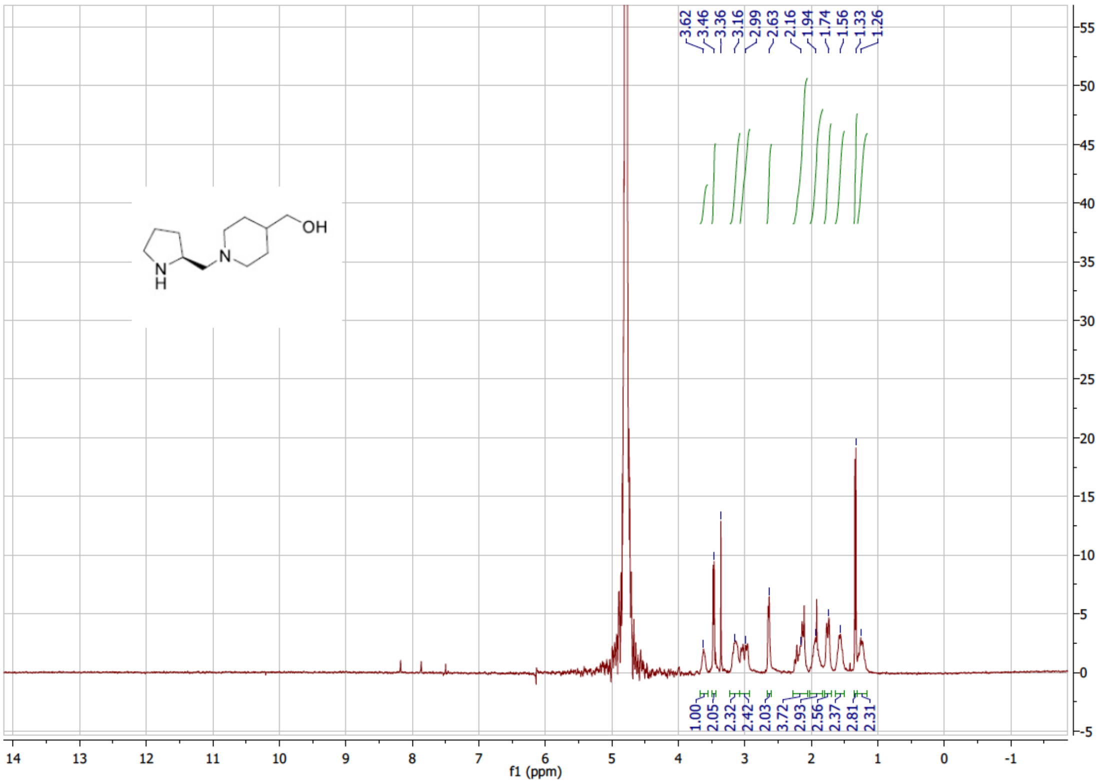

^{1}H NMR (400 MHz, D_2O)

HPLC traces for the Michael addition of cyclohexanone to 2-nitrostyrene
(Scheme 10 in the main text)

a) *rac*-**21**

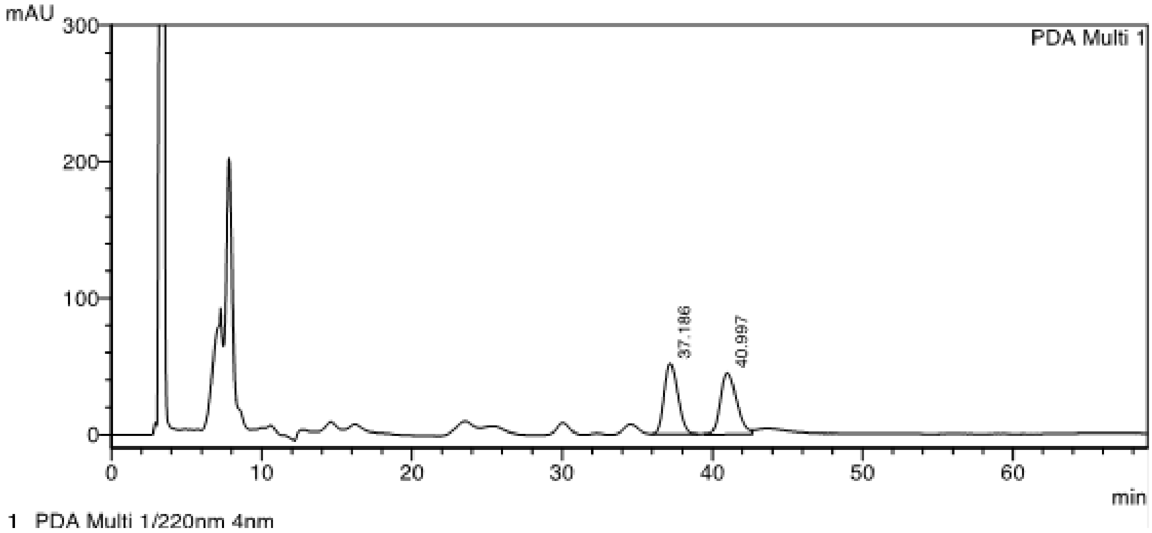

PeakTable
PDA Ch1 220nm 4nm

Peak#	Ret. Time	Area	Height	Area %	Height %
1	37.186	3298917	51734	49.331	53.484
2	40.997	3388417	44994	50.669	46.516
Total		6687334	96728	100.000	100.000

b) catalyst **13**

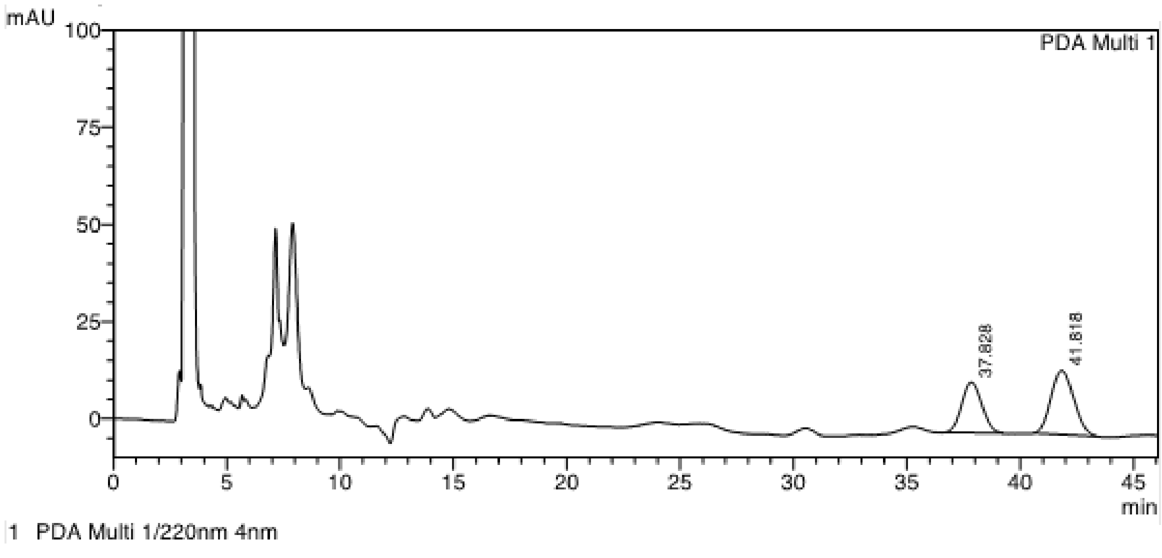

PeakTable
PDA Ch1 220nm 4nm

Peak#	Ret. Time	Area	Height	Area %	Height %
1	37.828	799812	12981	40.955	44.104
2	41.818	1153088	16452	59.045	55.896
Total		1952900	29434	100.000	100.000

c) catalyst **17**

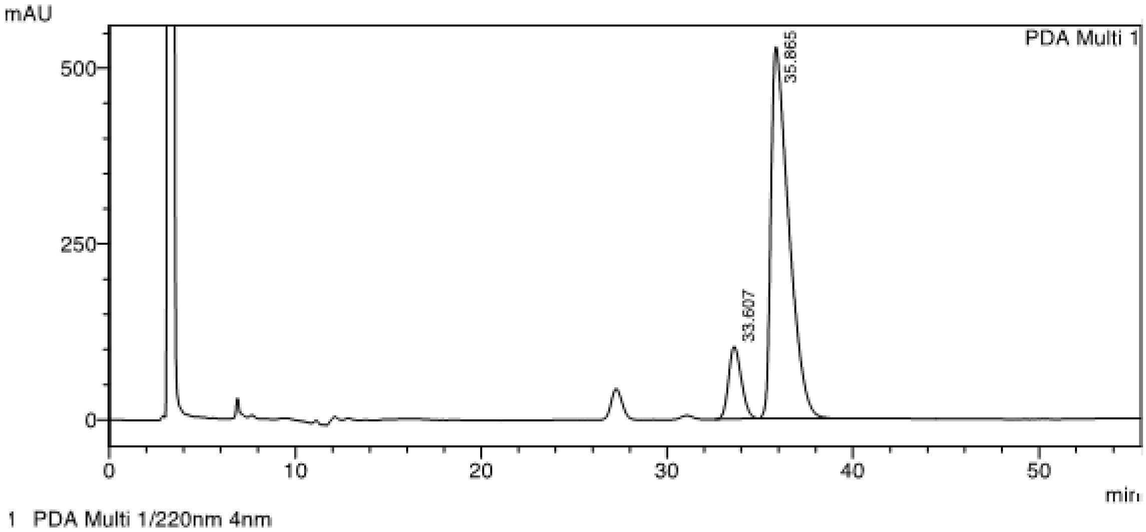

PeakTable

PDA Ch1 220nm 4nm

Peak#	Ret. Time	Area	Height	Area %	Height %
1	33.607	4893313	101753	12.197	16.151
2	35.865	35226569	528261	87.803	83.849
Total		40119882	630014	100.000	100.000

HPLC trace for the aldol addition of acetone to 4-benzaldehyde

(Scheme 9, Table 2 in the main text)

a) *rac*-**19**

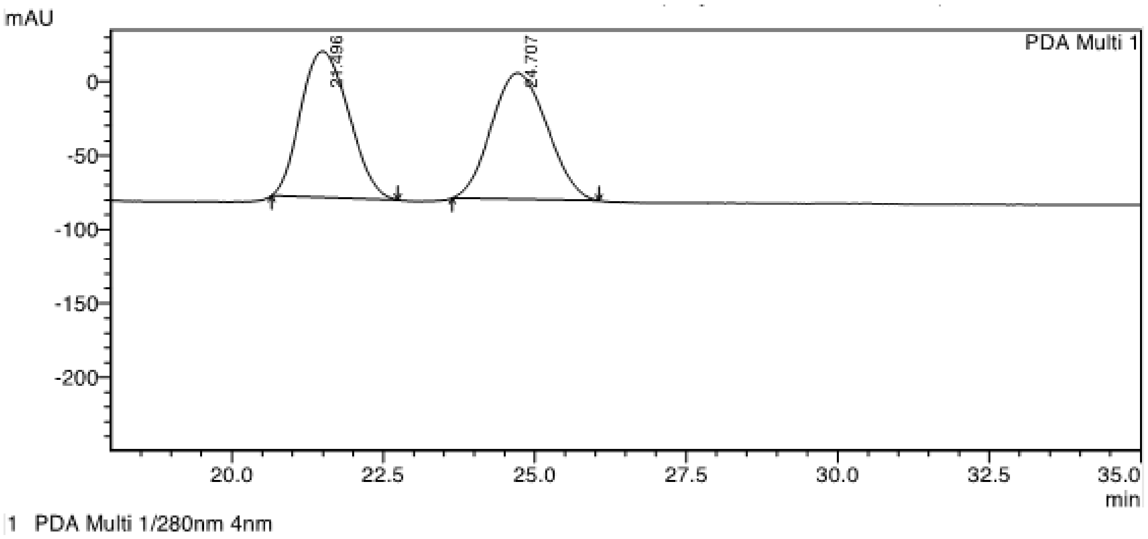

PeakTable

PDA Ch1 280nm 4nm

Peak#	Ret. Time	Area	Height	Area %	Height %
1	21.496	5451673	98643	50.017	53.674
2	24.707	5448007	85140	49.983	46.326
Total		10899680	183784	100.000	100.000

b) catalyst **13** (entry 4 in Table 2)

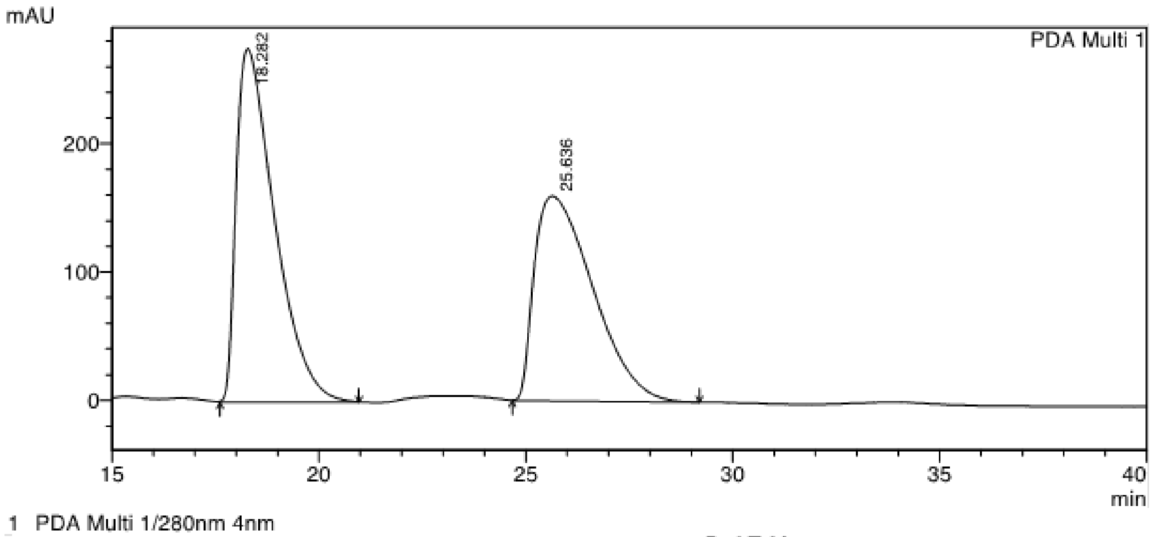

PeakTable

PDA Ch1 280nm 4nm

Peak#	Ret. Time	Area	Height	Area %	Height %
1	18.282	17861921.89	275550	53.13506	63.305
2	25.636	15754151.45	159727	46.86494	36.695
Total		33616073.34	435278	100.00000	100.000

HPLC traces for the aldol addition of cyclohexanone to 4-benzaldehyde

(Scheme 7, Table 1 in the main text)

a) *rac*-**16a** + *rac*-**16b**

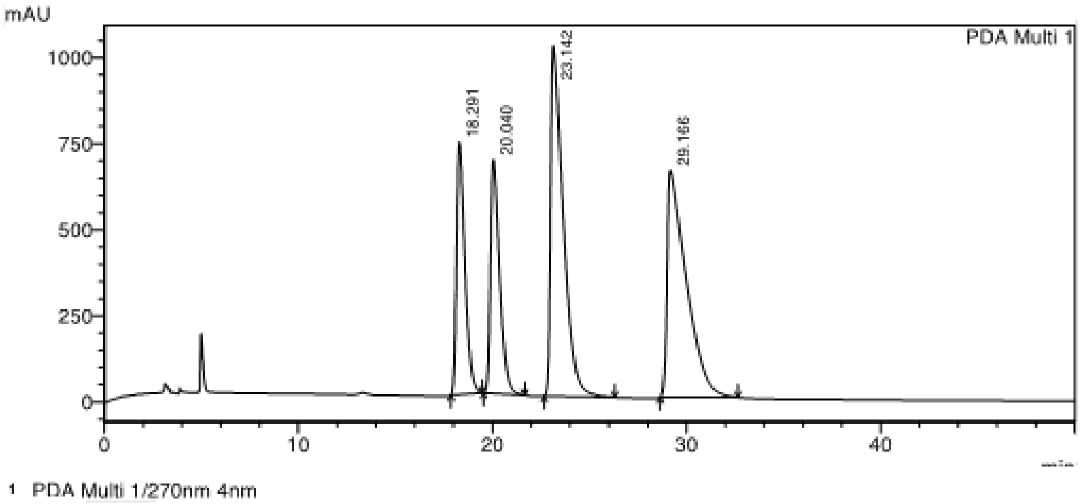

PeakTable

PDA Ch1 270nm 4nm

Peak#	Ret. Time	Area	Height	Area %	Height %
1	18.291	22366901	732436	16.392	23.710
2	20.040	22532458	679083	16.513	21.983
3	23.142	45982976	1018392	33.699	32.967
4	29.166	45568458	659205	33.396	21.340
Total		136450793	3089116	100.000	100.000

b) catalyst **8** (entry 3 in Table 1)

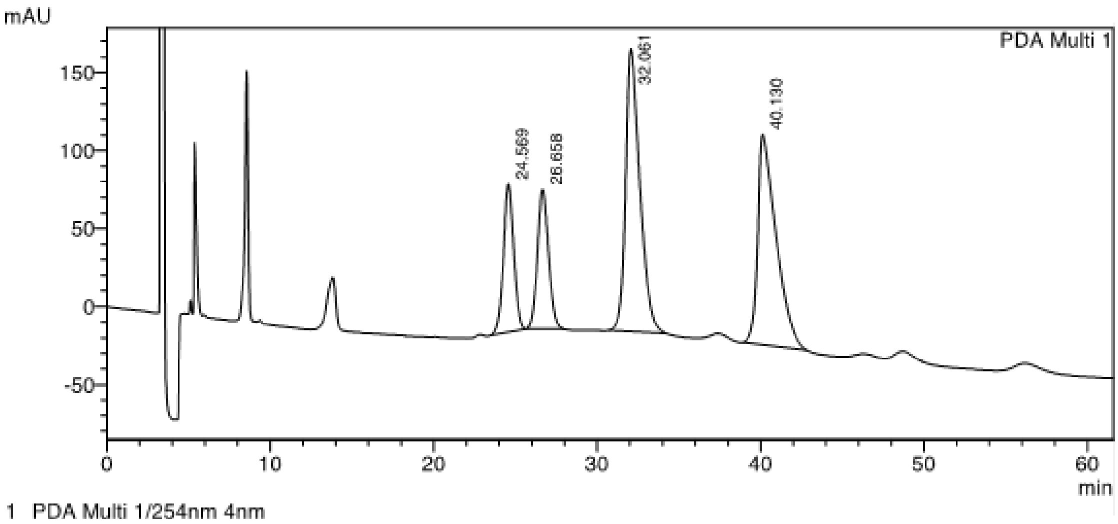

PeakTable

PDA Ch1 254nm 4nm

Peak#	Ret. Time	Area	Height	Area %	Height %
1	24.569	4146241	94832	13.845	18.958
2	26.658	4228352	89481	14.119	17.889
3	32.061	11001001	181091	36.735	36.203
4	40.130	10571555	134807	35.301	26.950
Total		29947149	500211	100.000	100.000

c) catalyst **13** (entry 4 in Table 1)

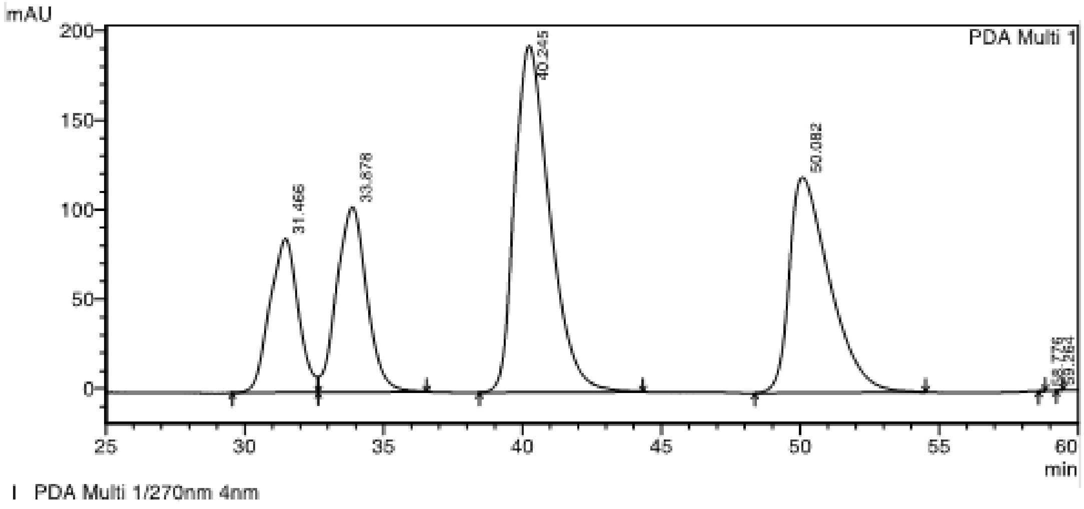

PeakTable

PDA Ch1 270nm 4nm

Peak#	Ret. Time	Area	Height	Area %	Height %
1	31.466	6125960.05	85956	14.19978	17.071
2	33.878	7779629.52	103374	18.03294	20.531
3	40.245	17057723.94	193613	39.53927	38.453
4	50.082	12175369.96	120336	28.22212	23.900
5	58.776	1309.64	112	0.00304	0.022
6	59.264	1233.56	117	0.00286	0.023
Total		43141226.68	503507	100.00000	100.000

d) catalyst **17** (entry 6 in Table 1)

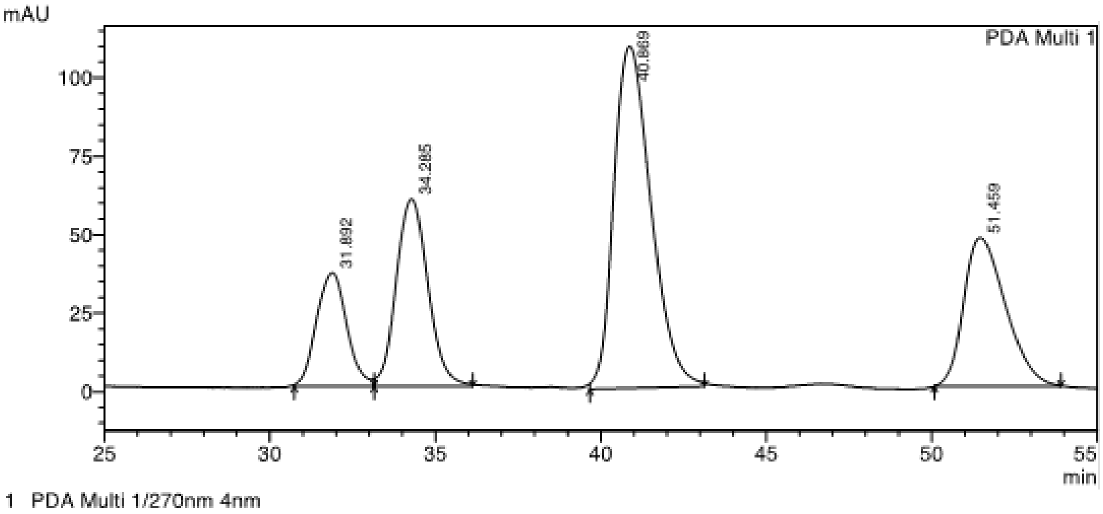

PeakTable

PDA Ch1 270nm 4nm

Peak#	Ret. Time	Area	Height	Area %	Height %
1	31.892	2296217.64	36078	12.11178	14.321
2	34.285	3977340.68	59645	20.97915	23.677
3	40.869	8515801.16	108927	44.91802	43.240
4	51.459	4169183.09	47265	21.99105	18.762
Total		18958542.57	251916	100.00000	100.000

e) catalyst **17** + *p*TsOH (entry 7 in Table 1)

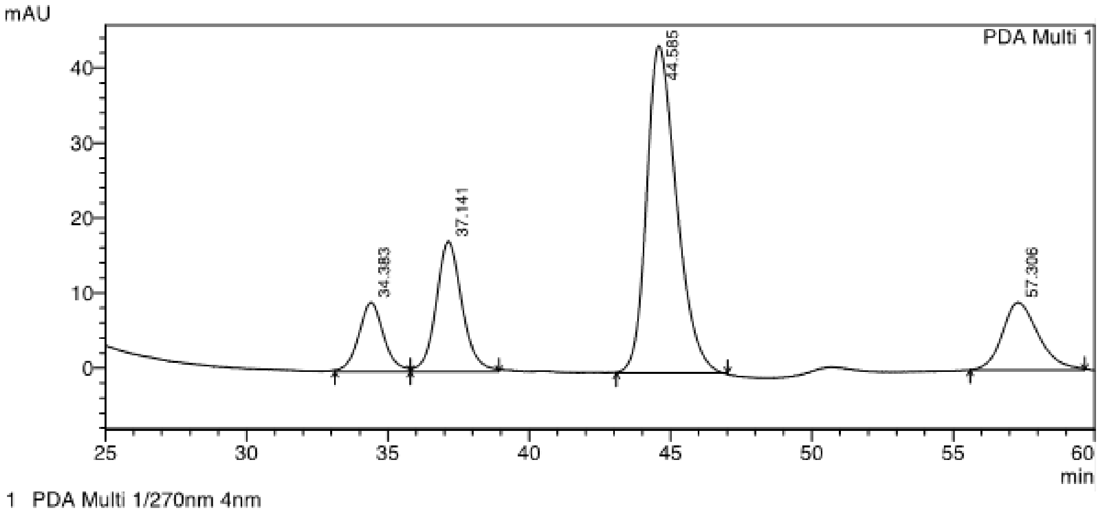

PeakTable

PDA Ch1 270nm 4nm

Peak#	Ret. Time	Area	Height	Area %	Height %
1	34.383	567262.91	9219	9.93791	11.641
2	37.141	1108369.20	17333	19.41759	21.887
3	44.585	3231745.52	43640	56.61715	55.106
4	57.306	800690.48	9001	14.02735	11.366
Total		5708068.10	79193	100.00000	100.000
